# Validity evidence for a novel instrument assessing medical student attitudes toward instruction in implicit bias recognition and management

**DOI:** 10.1186/s12909-021-02640-9

**Published:** 2021-04-12

**Authors:** Cristina M. Gonzalez, Joseph H. Grochowalski, Ramya J. Garba, Shacelles Bonner, Paul R. Marantz

**Affiliations:** 1 Department of Medicine, Albert Einstein College of Medicine, Bronx, NY USA; 2 Montefiore Medical Center- Weiler Division, 1825 Eastchester Road, DOM 2-76, Bronx, NY 10461 USA; 3 Fordham University, Bronx, NY USA; 4 University of Texas at Austin, Austin, TX USA; 5 Department of Emergency Medicine, Yale School of Medicine, New Haven, CT USA; 6 Departments of Epidemiology and Population Health and Medicine, Albert Einstein College of Medicine, Bronx, NY USA

## Abstract

**Background:**

Implicit bias instruction is becoming more prevalent in health professions education, with calls for skills-based curricula moving from awareness and recognition to management of implicit bias. Evidence suggests that health professionals and students learning about implicit bias (“learners”) have varying attitudes about instruction in implicit bias, including the concept of implicit bias itself. Assessing learner attitudes could inform curriculum development and enable instructional designs that optimize learner engagement. To date, there are no instruments with evidence for construct validity that assess learner attitudes about implicit bias instruction and its relevance to clinical care.

**Methods:**

The authors developed a novel instrument, the *Attitude Towards Implicit Bias Instrument* (ATIBI) and gathered evidence for three types of construct validity- content, internal consistency, and relationship to other variables.

**Results:**

Authors utilized a modified Delphi technique with an interprofessional team of experts, as well as cognitive interviews with medical students leading to item refinement to improve content validity. Seven cohorts of medical students, *N* = 1072 completed the ATIBI. Psychometric analysis demonstrated high internal consistency (*α* = 0.90). Exploratory factor analysis resulted in five factors. Analysis of a subset of 100 medical students demonstrated a moderate correlation with similar instruments, the Integrative Medicine Attitude Questionnaire (*r* = 0.63, 95% CI: [0.59, 0.66]) and the Internal Motivation to Respond Without Prejudice Scale (*r* = 0.36, 95% CI: [0.32, 0.40]), providing evidence for convergent validity. Scores on our instrument had low correlation to the External Motivation to Respond Without Prejudice Scale (*r* = 0.15, 95% CI: [0.09, 0.19]) and the Groningen Reflection Ability Scale (*r* = 0.12, 95% CI: [0.06, 0.17]) providing evidence for discriminant validity. Analysis resulted in eighteen items in the final instrument; it is easy to administer, both on paper form and online.

**Conclusion:**

The Attitudes Toward Implicit Bias Instrument is a novel instrument that produces reliable and valid scores and may be used to measure medical student attitudes related to implicit bias recognition and management, including attitudes toward acceptance of bias in oneself, implicit bias instruction, and its relevance to clinical care.

**Supplementary Information:**

The online version contains supplementary material available at 10.1186/s12909-021-02640-9.

## Introduction

Patients worldwide continue to report prejudice and bias in their clinical encounters [1–6]. Evidence suggests that implicit bias may be contributing to these disparate clinical experiences [7]. Implicit bias refers to the unconscious, unintentional mental associations we make based on social identity groups; it is a result in part, of systemic discrimination [8]. It is most commonly measured by the Implicit Association Test, a publicly available response latency test that pairs images and value laden words [9]. Implicit bias contributes to health disparities through its influence on provider communication patterns and clinical decision-making [10]. This evidence spans the spectrum of training and practice and is relevant to all health professions [7, 11–16].

Due to its contributions to health disparities, implicit bias is a focus of instruction in health professions education. Curricula have been published in undergraduate, graduate, and continuing health professions education [17–25]. These interventions have increased knowledge and awareness of implicit bias, with some achieving strategy identification to address it [17, 20]. Recently, enthusiasm for moving implicit bias instruction from increased awareness to skill-development and practice has emerged [26]. This approach facilitates learners developing skills to manage their biases in order to optimize the outcomes of their clinical encounters, a process called implicit bias recognition and management (IBRM) [27]. The outcome of skills-based IBRM instruction is behavioral, rather than a change in the IAT score [27]. Focusing on behaviors as the outcome of IBRM instruction would obviate the limitations of the IAT [28].

Attitudes influence behaviors. Qualitative explorations of students’ attitudes have at times demonstrated resistance to the existence of implicit bias in general, its presence within oneself, its relevance to clinical care, and other aspects of instruction [17, 26, 29]. Physicians, nurses, and residents, struggle with reconciling their implicit bias with their professional identities [30, 31]. We have demonstrated the detrimental effect of learner resistance on faculty perceptions of their ability to facilitate implicit bias instruction [32]. Our previous work expanded on what is known about student resistance revealing potential threats to engagement with IBRM instruction, as well as opportunities to maximize engagement [26].

Better understanding attitudes about implicit bias could inform curriculum development. To our knowledge, no validated instrument exists to assess learner attitudes toward acceptance of bias in oneself, implicit bias instruction, or its relevance to clinical care (henceforth collectively referred to as IBRM instruction). Without a validated instrument to assess these attitudes, comprehensive curriculum development and program evaluation related to IBRM will remain an elusive goal. To address this gap, we designed and obtained evidence for construct validity of a novel instrument assessing student attitudes about IBRM instruction.

## Methods

We developed the Attitude Toward Implicit Bias Instrument (ATIBI) through a series of steps to assess three types of validity: content, internal structure, and relationship to other variables [33]. We followed the instrument validation processes and methodology that have been established in related literature on attitude measurement in clinical education [34–36]. The study and all methods and procedures were reviewed and approved by the Institutional Review Board (IRB) of the Albert Einstein College of Medicine; it was deemed exempt research, no written consent was required.

### Construct validity- content

#### Item design

The initial item design began with a literature search (CMG) of PubMed, ERIC, PsycNET, and Google Scholar using the terms “implicit bias” or “unconscious bias” or “subconscious bias” and “attitudes” to identify any existing instruments in the winter of 2015. No existing validated instruments were identified. We generated initial survey items among our team (CMG, RJG, PRM) informed by three sources: 1) Prior survey design at our institution [18]; 2) Initial qualitative data analysis of medical students’ perceptions of challenges and opportunities for participating in implicit bias instruction [26]; and 3) Lessons learned from our experiences delivering instruction on implicit bias [18, 20]. After two rounds of revisions, we convened a panel of experts to participate in a modified Delphi technique in the spring of 2015; the Delphi was considered modified as the group interacted during the two pre-determined rounds [37]. In the spring of 2015, experts included three cognitive psychologists, and four clinician-investigators (one nurse and three physicians), all with previous experience in implicit bias education. We sent the initial survey items out for review, with the option to provide written feedback prior to the initial meeting. The meeting was held via web conference, each item was reviewed, accepted, reworded, or discarded. Additional items were suggested. Items were sent out for another round of feedback. During the second and final meeting, we reviewed each item and achieved consensus through discussion. We also agreed to a six-item Likert-type scale with anchors ranging from strongly disagree to strongly agree; we chose an even-numbered scale to avoid a neutral option, given our focus on learner attitudes about this topic.

#### Item refinement

In order to refine the final items on the scale, we recruited a convenience sample of 20 medical student volunteers from Albert Einstein College of Medicine in Bronx, NY, USA. Students completed the instrument and participated in cognitive interviews for each question in the summer of 2015. During the cognitive interviews, investigators read each survey item with the medical student volunteers and discussed their interpretation of it, with the possibility of rewording for clarification. Cognitive interviews are a methodology to improve survey construct validity due to their exploratory nature; they reveal reasons for respondents’ answers, and identify which questions may be being interpreted differently than investigators intend [38]. Each student received a $25 gift card as compensation for their time.

#### Administration

The full scale was administered to six separate cohorts of students from 2015 to 2018. Four cohorts of first-year medical students completed a confidential, online survey prior to a novel session on implicit bias introduced during orientation week. Two cohorts of third-year medical students, distinct students from the first-year cohorts, completed the same survey on paper prior to a required session on implicit bias (2016–2017). Although the third-year students were further along in their medical education, all participants took the survey prior to being exposed to any formal instruction during medical school related to IBRM. Item responses (e.g., “strongly agree”, “slightly disagree”) were assigned integer values; a student’s score was a sum of response values for all items. For each administration, a cover sheet was included explaining the voluntary nature of the study and providing information on its IRB approval.

### Construct validity- internal structure

We examined evidence for item cohesion and internal structure using psychometric measures including reliability, item-total and item-rest correlations, and assessed the factor structure using exploratory factor analysis with correlated factor scores. The purpose of this analysis was to determine how well the items measured the ATIBI construct(s), and whether there was enough cohesion among items and sufficient variability in the ATIBI scores to reliably distinguish between persons with different scores. The item-total correlation is the correlation of the item’s score with the total score, which reflects how strongly the item measures the overall construct. Standard correlation is the correlation of the item with the total score if all items were standardized. The item-rest score is the item’s score correlated with the total score from the scale, minus the item’s score. This provides information about how strongly the item measures the construct while not influencing the total score. The analysis of scores and scale structure was conducted using the R Psych package [39].

### Construct validity: relationship to other variables

A second convenience sample of medical student volunteers completed four additional scales in the spring of 2017. These students were a sub-set of volunteers who had taken the ATIBI and responded to an invitation to take the additional scales afterward. The first 100 students who responded were eligible. Each student received a $25 gift card as compensation for their time. To assess for convergent validity, we correlated scores between the ATIBI and four other measures. The Integrative Medicine Attitude Questionnaire (IMAQ) was selected as a convergent scale; it includes factors related to openness to new ideas, paradigms, and interactions between patients and providers [40]. It has been administered to medical students, trainees, and practicing physicians (*α* = 0.89) [40]. Implicit bias instruction requires learners to be open to recognizing biases about which they may have not been previously aware. In addition, in our previous work, we identified opportunities to restore patient-provider rapport should bias be perceived during an encounter, which requires an openness and attention to the dynamics of the patient-provider interaction [6]. The second convergent scale selected was the Internal Motivation to Respond Without Prejudice Scales (IMS); it measures participants personal, internal motivations regarding prejudice, and implicit bias is internally accepted and personal as well [41]. It has been administered to university students (*α* = 0.83) [41]. The External Motivation to Respond Without Prejudice Scale (EMS) and the Groningen Reflection Ability Scale (GRAS) were selected as a discriminant scales. EMS measures response to external pressures regarding prejudiced responses; it has been administered to university students (*α* = 0.78) [41]. GRAS measures self-perceived personal reflection ability, as a general skill in professional growth; it has been administered to medical students (*α* = 0.83) [42].

We estimated convergent and discriminant validity coefficients for the scale using a Multitrait-Multimethod matrix (MTMM), using the R base package [39]. The MTMM method for inspecting validity does not have any direct standards for interpretation. In MTMM, constructs that are expected to be similar (i.e., convergent) should have larger correlations with the construct of interest, while constructs that are expected to be dissimilar should have smaller correlations [43, 44]. In their textbook on psychometric theory, Raykov and Marcoulides [45] affirm this approach, saying that convergent validity measures should be lower than the construct’s (i.e., ATIBI) reliability, but higher than the discriminant validity coefficients: “This is consistent with theoretical expectations, given the convergent validity coefficients reflect relationships between different measures of the same trait, whereas the discriminant validity coefficients reflect considerably weaker relationships between different indicators of different constructs” [page 222]. Therefore, we expected the convergent IMAQ and IMS scores to have larger correlations with the ATIBI than the EMS and GRAS scores, which are discriminant.

**Fig. 1:**
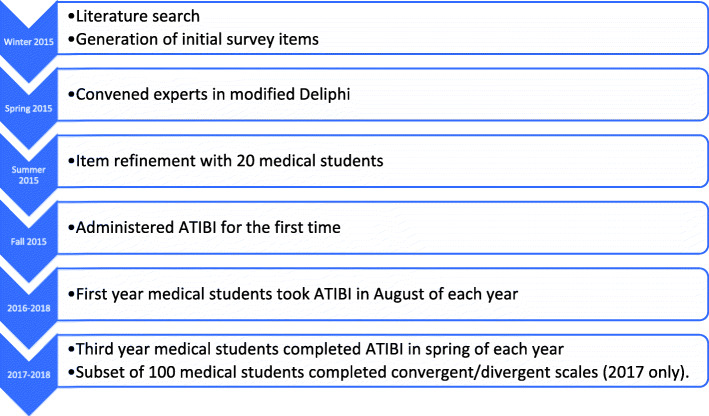
Timeline of all procedures for development of Attitudes Toward Implicit Bias Instrument (ATIBI) at Albert Einstein College of Medicine, New York, USA. Psychometric analysis occurred iteratively throughout

A timeline of all procedures is outlined in Fig. 1.

## Results

### Construct validity: content

#### Item design

Through our Delphi technique, we developed a 27-item scale. This was created from 44 items originally identified by the authors, 24 items after the first round and three additional items resulting from the second round with the expert panel. The finalized items are reported in Table 1. The expert panel determined it was imperative to include questions related to the following constructs: 1) implicit bias as a valid concept; 2) implicit bias existing within oneself; 3) the potential for implicit bias to influence clinical care; 4) the value of implicit bias in medical student education; and 5) their self-perceived confidence level in recognizing and managing one’s own implicit biases. As items were reviewed, accepted, reworded, or discarded, and additional items suggested, participants ensured that the five constructs were represented within the evolving questions.

**Table 1:** Items on the original administration of the Attitudes Toward Implicit Bias Instrument after expert consensus through modified Delphi-technique, 2015–2018

1. (When I make an assumption about someone different than me, the person overreacts.) 2. Individuals carry assumptions and opinions in their subconscious (in the form of implicit bias) that they are not aware of. 3. Racial and ethnic minority groups are often treated in subtly disrespectful ways. 4. Learning about implicit bias is as important to the practice of medicine as learning about basic science. 5. It is important to me to learn how to recognize when one of my own implicit biases is activated. 6. I am able to define implicit bias in my own words. 7. The personal implicit biases that other students hold about racial and/or ethnic minorities may affect the quality of care they provide to patients. 8. I worry about saying the wrong thing during discussions about racial and ethnic implicit bias. 9. Implicit bias recognition and management is a competency students should master before attaining their medical degree 10. I have made assumptions about racial and/or ethnic minorities that have proven to be incorrect 11. I worry that my actions won’t match my values when I interact with patients who are racially or ethnically different than me. 12. (The personal implicit biases that I myself hold about racial and/or ethnic minorities may affect the quality of care I provide to patients.) 13. Racism is only an issue of the past. 14. It is important to me to learn how to minimize the effects my implicit biases may have on my clinical decision-making.	15. (Medical schools have a responsibility to help students become aware of their biases and their potential impact on clinical decision making.) 16. Learning about implicit bias is as important to the practice of medicine as learning about patient-physician communication skills. 17. When I have an exam looming I don’t want to waste time learning about implicit bias. 18. I have the skills to address my own implicit biases that come up in the course of delivering care. 19. An individual’s implicit bias can affect her/his/their behavior. 20. (Learning about implicit bias is as important to the practice of medicine as learning about clinical reasoning.) 21. The assumptions I make about racial and/or ethnic minorities may affect the way I treat them 22. It is important to discuss race, ethnicity, and culture during medical school. 23. (Implicit bias class discussions should allow for all opinions to be expressed) 24. (The US health care system provides fair and equitable care to all populations, regardless of their race, ethnicity and/or immigration status) 25. (The personal implicit biases physicians hold about racial and/or ethnic minorities may affect the quality of care they provide to patients.) 26. (I feel comfortable during discussions about race and ethnicity.) 27. (If a test were to find that I subconsciously favor one racial or ethnic group over another, I would question the validity of the test)

Note: Items in parentheses were subsequently removed because of poor item statistics or low construct loadings

#### Item refinement

Upon completion of the ATIBI, we discussed each item with students in the initial cohort (*N* = 13). Three items were reworded. A second cohort of students took the ATIBI with the reworded items (*N* = 7). One reworded item was still confusing, and we eliminated it completely from our scale, leaving 26 items for the internal structure analysis.

### Construct validity: internal structure

#### Sample

A total of 1281 students were eligible to participate. Students who were absent from the instructional session, could not connect via wireless internet to the survey, or for whom we had incomplete survey data were excluded, leading to a final sample of 705 first-year and 367 third-year students, for a total of *n* = 1072 (84% response rate). Student demographic data are presented in Table 2. There were no statistically significant differences between demographic groups (all *p* values ≥0.05). However, there was a significant overall difference of 3.7 points (*t* = 4.8(688.86), *p* < .001, 95% CI = [2.2, 5.2]) between first-year students (MN = 90.9, SD = 10.8) and third-year students (MN = 87.2, SD = 12.9).

**Table 2:** Average total scores on Attitude Toward Implicit Bias Instrument by demographic characteristics and year in medical school. Students completed the instrument between 2015 and 2018 in New York, USA

Variable	Year	Category	N	Mean	s.d.
Latino/a/x	1st	Latino/a/x	52	92.6	9.8
	1st	Not Latino/a/x	646	90.8	10.8
	3rd	Latino/a/x	16	87.6	13.2
	3rd	Not Latino/a/x	286	87.7	13.1
Race	1st	African American/Black	25	94.5	6.7
	1st	Asian/South Asian/Pacific Islander	217	93.9	9.6
	1st	White	408	89.2	11.0
	1st	Mixed	39	91.0	11.9
	1st	Other	11	89.4	9.8
	3rd	African American/Black	11	89.9	15.0
	3rd	Asian/South Asian/Pacific Islander	69	86.3	11.5
	3rd	White	225	86.5	13.3
	3rd	Mixed	4	83.3	16.3
	3rd	Other	13	82.8	12.5
Gender	1st	Female	338	93.3	9.4
	1st	Male	358	88.7	11.4
	1st	Other	4	97.0	7.8
	3rd	Female	171	90.5	12.1
	3rd	Male	194	84.3	13.2
Orientation	1st	Bisexual	20	96.3	8.2
	1st	Heterosexual	602	90.4	10.9
	1st	Homosexual	31	93.0	10.1
	1st	Queer	7	98.6	7.3
	1st	Other	36	93.9	7.6
	3rd	Bisexual	8	91.3	14.9
	3rd	Heterosexual	283	89.6	13.0
	3rd	Homosexual	20	89.6	8.8
	3rd	Queer	5	100.0	5.1
	3rd	Other	27	89.5	13.2
	3rd	(Missing)	24	80.7	13.4
Age	1st	< 25	579	90.7	10.7
	1st	25–30	101	91.7	11.1
	1st	> 30	19	94.1	8.7
	3rd	< 25	88	87.7	12.0
	3rd	25–30	259	87.4	13.3
	3rd	> 30	16	81.9	14.1

#### Item and reliability analysis

Although the initial reliability of the scores was high (*α* = 0.90), three items were removed from the scale after administration to the first cohort (*N* = 155 in 2015) because their scores had low correlations with the total scores, suggesting they did not measure the implicit bias construct strongly enough to keep in the questionnaire. This survey (Additional file 1) underwent further testing with a larger cohort from 2016 to 2018, another five items were removed because of low item-total correlations and no contribution to the measured factor structure. The final ATIBI contained 18 items, each scored on a scale of 1 to 6, resulting in a total possible score range of 18 to 108, with higher scores reflecting more positive attitudes. This 18-item survey was administered to the subsequent cohorts. The scores had very high reliability (*α* = 0.90), suggesting that responses to items across the scale are consistent. The mean score was 89.4 (out of 108) with a standard deviation of 11.7. The standard error of measurement was estimated to be 3.7, indicating a relatively small amount of measurement error. All items in general had strong correlations. The average item score and standard deviation, the item-total correlation, standard correlation and item-rest correlation (RD) are included in Table 3.

**Table 3:** Classical item statistics for items included in the final Attitudes Toward Implicit Bias Instrument

Item^a^	N	Item-total correlation	Standard correlation	Item-rest correlation	Mean	s.d.
2	950	0.56	0.56	0.52	5.5	0.71
3	950	0.66	0.66	0.62	5.2	0.90
4	950	0.73	0.71	0.67	5.0	1.24
5	950	0.73	0.75	0.70	5.5	0.74
6	950	0.73	0.74	0.70	5.4	0.80
7	950	0.70	0.70	0.66	5.1	0.98
8	950	0.54	0.49	0.47	4.5	1.10
9	950	0.50	0.41	0.40	3.6	1.44
10	950	0.60	0.53	0.52	3.9	1.47
11	950	0.36	0.32	0.29	5.6	0.88
13	950	0.75	0.76	0.71	5.3	0.85
14	950	0.73	0.72	0.68	5.0	1.16
16	950	0.68	0.70	0.65	5.4	0.74
17	950	0.73	0.71	0.66	4.4	1.46
18	950	0.63	0.58	0.56	4.6	1.27
19	950	0.70	0.70	0.66	5.4	0.77
21	950	0.42	0.36	0.33	4.8	1.26
22	950	0.73	0.73	0.69	5.2	0.82

^a^Item numbers refer to their location in Table 1

#### Factor analysis

**Fig. 2:**
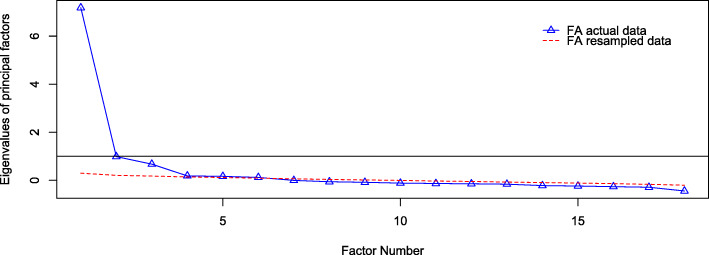
Scree plot of factor analysis eigenvalues (vertical axis) against the factor numbers (horizontal axis). The solid line marks eigenvalues greater than 1 and the dotted line marks eigenvalues that are greater in value than resampled data that has no factor structure

An exploratory factor analysis of the 18-item scale (using correlated factor scores via Oblimin rotation in the R Psych package) strongly suggested five factors (based on parallel analysis results depicted in the screen plot in Fig. 2) underlie the responses to items on the ATIBI (RMSEA = 0.043, TLI = 0.97). The factor loadings and proportions of variance explained by the factors are in Table 4. The first factor, labeled Valuing Implicit Bias Instruction in Medical Education based on the pattern of item loadings, explained 14% of the total variance in the data. The second factor, Acceptance of Implicit Bias in Oneself, explained 11%, the third, Self-Awareness/Perceived Self-Efficacy related to implicit bias, explained 13%, the fourth, Recognition of the Importance of Implicit Bias, explained 12%, and the last, Acceptance of the Impact of Implicit Bias on Clinical Care, explained 4%. In total, 54% of the total variability in the data was explained by the factor structure. Table 4 also includes the communal variance, which is the proportion of each item’s variance that was accounted for by the factors. Table 5 lists the factor correlations, which are the correlations between scores on each of the factors; all factors have low-to-moderate intercorrelations, suggesting a single common higher-order factor is present.

**Table 4:** Factor loadings and variance explained from an exploratory factor analysis of Attitudes Toward Implicit Bias Instrument items

		Factor					
	Item Number^a^	1	2	3	4	5	Communal Variance
Item Loadings	Item 2	–	–	0.64	–	–	0.48
	Item 3	–	–	0.40	–	0.30	0.52
	Item 4	0.74	–	–	–	–	0.72
	Item 5	–	–	0.46	0.37	–	0.66
	Item 6	–	–	0.58	–	–	0.62
	Item 7	0.30	–	–	0.43	–	0.61
	Item 8	–	0.43	–	–	–	0.36
	Item 9	–	0.74	–	–	–	0.54
	Item 10	–	0.80	–	–	–	0.69
	Item 11	–	–	–	–	0.39	0.23
	Item 13	–	–	–	0.77	–	0.73
	Item 14	0.67	–	–	–	–	0.72
	Item 16	–	–	0.48	–	–	0.60
	Item 17	0.93	–	–	–	–	0.84
	Item 18	–	0.53	–	–	–	0.59
	Item 19	–	–	–	0.65	–	0.61
	Item 21	–	–	–	–	0.57	0.35
	Item 22	–	–	0.40	–	–	0.62
Proportion of Variance Explained		0.14	0.11	0.13	0.12	0.04	

^a^Item numbers refer to their location in Table 1

**Table 5:** Inter-correlations between factors within the Attitudes Toward Implicit Bias Instrument

	F1	F2	F3	F4	F5
F1: Valuing Implicit Bias Instruction	1				
F2: Acceptance of Implicit Bias in Oneself	0.33	1			
F3: Self-Awareness/Perceived Self-Efficacy	0.49	0.40	1		
F4: Recognition of the Importance	0.73	0.35	0.62	1	
F5: Acceptance of the Impact on Clinical Care	0.27	0.24	0.47	0.39	1

### Construct validity: relationship to other variables

The ATIBI scores were moderately convergent with the IMAQ (*r* = 0.63, 95% CI: [0.59, 0.66]) and IMS (*r* = 0.36, 95% CI: [0.32, 0.40]). This suggests that a small-but-not-insignificant proportion of the variability in the respective scores are related, suggesting that they measure some overlapping information, providing evidence for convergent validity. The ATIBI scores had lower correlations with the EMS (*r* = 0.15, 95% CI: [0.09, 0.19]) and GRAS (*r* = 0.12, 95% CI: [0.06, 0.17]), suggesting that the scores measure discriminant constructs, providing evidence for discriminant validity. Note that these correlations match the expected pattern described by Sawilowsky [44] and Raykov & Marcoulides [45], that the ATIBI reliability (*α* = 0.90) should be greater than the convergent correlations (0.63, 0.36), which should be in turn greater than the discriminant correlations (0.15, 0.19).

## Discussion

We describe the development and provide evidence for construct validity of the Attitudes Toward Implicit Bias Instrument (ATIBI), a novel survey instrument to assess learner attitudes toward IBRM instruction. Given the efforts across health professions to address implicit bias through curricular innovation, this survey instrument is both timely and significant. We believe the ATIBI has several strengths. To our knowledge, it is the first survey assessing learner attitudes regarding IBRM instruction that provides evidence of three forms of construct validity: content, internal structure, and relationship to other variables. The Delphi expert panel consensus and the results of the cognitive interviews conducted with students provided strong evidence of content validity. The ATIBI shows evidence of construct validity related to internal structure with high reliability scores. The exploratory factor analysis suggests subscales in attitudes toward the importance of implicit bias instruction (both in general and with specific attention to recognition and management), acceptance of the presence of bias in oneself, its impact on clinical care, and recognition of systemic discrimination. Finally, the ATIBI shows evidence of construct validity for relation to other variables, being able to discriminate among similar and dissimilar constructs. The ATIBI is easy to administer electronically or on paper, and in our experience, it should take learners approximately 10 min or less to complete.

Transformative learning theory (TLT) has been proposed as an effective guide for instruction in IBRM [27]. TLT can challenge existing attitudes and facilitate a questioning of those attitudes leading to eventual paradigm shifts [46]. Briefly, TLT has four parts, an experience, critical reflection, dialogue, and behavior change [46]. The experience can be created for the learners and should be profound, a “disorienting dilemma,” in order to engage learners through the remaining phases of TLT [46, 47]. For sensitive topics such as racial bias, it is important to meet learners where they are in terms of their attitudes [48]. The ATIBI, therefore, could serve two purposes to enhance efforts in curriculum development for IBRM: 1) the ATIBI could serve as a baseline assessment of learner attitudes, thereby informing curriculum development, including the experience aspect of TLT; and 2) in aggregate it could inform program evaluations of components of curricula that target learner attitudes toward IBRM potentially identifying successful components of the curriculum and those parts requiring revision. Future uses of the ATIBI include opportunities to evaluate the instrument’s ability to assess individual learner changes over time and to use the subscales in program evaluations to evaluate specific components of curricula.

## Limitations

Our study has some limitations. It is a single institution study and may not be fully generalizable across institutions (although we engaged experts in other institutions as part of our Delphi approach). Medical students at other institutions may have different attitudes. Given attitudes are self-reported, there exists the potential for social desirability bias. The item and reliability analysis are sample dependent, not all administrations of the ATIBI will return the same item and reliability statistics. Even in our own sample, the first- and third-year cohorts scored differently. We speculate that there was increasing popularity of implicit bias instruction in undergraduate education at the time, scores could be influenced by online versus paper administration, or it may be related to the differences in experience in clinical contexts between first- and third- years. Future research will endeavor to uncover the reason(s). The ATIBI was administered once for this analysis. Future efforts should evaluate its utility as a longitudinal instrument. Implicit bias is ubiquitous across health professions, and we only surveyed medical students. More research is needed to see if the instrument retains evidence for construct validity when adapted for other health professions students. Future analyses can include assessment of measurement invariance across populations (nurses, physician assistants, etc.) and confirmatory factor analysis for new cohorts to ensure the stability of psychometric coefficients over different samples. Although the items measure five factors, six or fewer items measure these constructs, which means that subscores on the five constructs measured by so few items would have very low reliability. For this reason, until further research is conducted, we are only able to suggest using the total ATIBI sum score, which is supported by the common factor in the exploratory factor analysis and the classical item statistics.

## Conclusion

In conclusion, the Attitudes Toward Implicit Bias Instrument is a novel instrument that produces reliable and valid scores and may be used to measure medical students’ attitudes toward acceptance of implicit bias in oneself, implicit bias instruction, and its relevance to clinical care.

## Supplementary Information

**Additional file 1.** Implicit bias attitude scale.

## Data Availability

Available from corresponding author upon request.
