# Supplementary material for: Validity evidence for a novel instrument assessing medical student attitudes toward instruction in implicit bias recognition and management
Source: BMC Med Educ. 2021 Apr 12;21:205. doi: 10.1186/s12909-021-02640-9 (PMC8040240; doi:10.1186/s12909-021-02640-9)
Supplement: Supplementary file 1 — Additional file 1. Implicit bias attitude scale. [file 12909_2021_2640_MOESM1_ESM.docx]

**Implicit Bias Attitude Scale**

1 = Strongly Disagree

2 = Disagree

3 = Slightly Disagree

4 = Slightly Agree

5 = Agree

6 = Strongly Agree

***IMPORTANT*: PLEASE WRITE DOWN THE LAST FIVE DIGITS OF YOUR BANNER ID#: ____________**

**1. I am able to define implicit bias in my own words.**

**1 2 3 4 5 6**

**2. Individuals carry assumptions and opinions in their subconscious (in the form of implicit bias) that they are not aware of.**

**1 2 3 4 5 6**

**3. Racial and ethnic minority groups are often treated in subtly disrespectful ways.**

**1 2 3 4 5 6**

**4. Learning about implicit bias is as important to the practice of medicine as learning about basic science.**

**1 2 3 4 5 6**

**5. It is important to me to learn how to recognize when one of my own implicit biases is activated.**

**1 2 3 4 5 6**

**6. The personal implicit biases that other students hold about racial and/or ethnic minorities may affect the quality of care they provide to patients.**

**1 2 3 4 5 6**

**7. Implicit bias recognition and management is a competency students should master before attaining their medical degree**

**1 2 3 4 5 6**

**8. I have made assumptions about racial and/or ethnic minorities that have proven to be incorrect**

**1 2 3 4 5 6**

**9. I worry that my actions won’t match my values when I interact with patients who are racially or ethnically different than me.**

**1 2 3 4 5 6**

**10. The personal implicit biases that I myself hold about racial and/or ethnic minorities may affect the quality of care I provide to patients.**

**1 2 3 4 5 6**

**11. Racism is only an issue of the past.**

**1 2 3 4 5 6**

**12. It is important to me to learn how to minimize the effects my implicit biases may have on my clinical decision-making.**

**1 2 3 4 5 6**

**13. Medical schools have a responsibility to help students become aware of their biases and their potential impact on clinical decision-making.**

**1 2 3 4 5 6**

**14. Learning about implicit bias is as important to the practice of medicine as learning about patient-physician communication skills.**

**1 2 3 4 5 6**

**15. I have the skills to address my own implicit biases that come up in the course of delivering care.**

**1 2 3 4 5 6**

**16. An individual’s implicit bias can affect her/his/their behavior.**

**1 2 3 4 5 6**

**17. Learning about implicit bias is as important to the practice of medicine as learning about clinical reasoning.**

**1 2 3 4 5 6**

**18. The assumptions I make about racial and/or ethnic minorities may affect the way I treat them**

**1 2 3 4 5 6**

**19. It is important to discuss race, ethnicity, and culture during medical school.**

**1 2 3 4 5 6**

**20. Implicit bias class discussions should allow for all opinions to be expressed**

**1 2 3 4 5 6**

**21. The US health care system provides fair and equitable care to all populations, regardless of their race, ethnicity and/or immigration status.**

**1 2 3 4 5 6**

**22. The personal implicit biases physicians hold about racial and/or ethnic minorities may affect the quality of care they provide to patients.**

**1 2 3 4 5 6**

**23. I feel comfortable during discussions about race and ethnicity.**

**1 2 3 4 5 6**

**24. My ethnicity is (Note: we are using the US Census-based approach, where “Hispanic ethnicity” is recorded as a separate axis from race):**

- - **Hispanic Or Latino**
  - **Not Hispanic or Latino**
  - **25. My race is:**
  - **African American/Black**
  - **Asian/South Asian/Pacific Islander**
  - **Native American**
  - **White**
  - **Other**
  - **26. My Gender is (choose all that apply):**
  - **Female**
  - **Male**
  - **Transgender (F to M)**
  - **Transgender (M to F)**
  - **Other:**
  - **27. My sexual orientation is:**
  - **28.      The number of years I spent between finishing college and starting medical school was:**
  - **0 (I went directly to med school)**
  - **1-2 years**
  - **3-5 years**
  - **>5 years**
  - **29. Age:**
  - **<25**
  - **25-30**
  - **>30**
  - **30. My undergraduate degree is in:**
  - **Physical Sciences**
  - **Biological Sciences**
  - **Social Sciences**
  - **Humanities**
  - **31. Other degrees I hold (write in):**
  - **32. My medical specialty of interest is (write in):**
  - **33.      The highest level of education my parent(s) attained is**
  - **< High school**
  - **HS Diploma**
  - **Associate's Degree**
  - **Bachelor's Degree**
  - **Master's Degree**
  - **Doctoral Degree**

**34. Primary Language (write in):**
